# A Structural Model of Teacher Self-Efficacy, Emotion Regulation, and Psychological Wellbeing Among English Teachers

**DOI:** 10.3389/fpsyg.2022.904151

**Published:** 2022-06-30

**Authors:** Shen Xiyun, Jalil Fathi, Naser Shirbagi, Farnoosh Mohammaddokht

**Affiliations:** ^1^School of Education, Lanzhou City University, Lanzhou, China; ^2^Department of English and Linguistics, Faculty of Language and Literature, University of Kurdistan, Sanandaj, Iran; ^3^Department of Education, Faculty of Humanities and Social Sciences, University of Kurdistan, Sanandaj, Iran

**Keywords:** teacher self-efficacy, emotion regulation, psychological wellbeing, EFL teachers, SEM

## Abstract

Because of the exacting nature of teaching, identifying factors affecting teachers’ mental health and psychological wellbeing are of paramount importance. Parallel with this line of inquiry, the goal of this project was to test a model of psychological wellbeing based on teacher self-efficacy and emotion regulation in an EFL context. To this end, 276 Iranian English teachers participated in this survey. First, the measurement models for the three latent constructs were verified through performing Confirmatory Factor Analysis (CFA). Then Structural Equation Modeling (SEM) was used to test the hypothesized model. SEM outcomes evince that both teacher self-efficacy and emotion regulation were the significant predictors of teachers’ psychological wellbeing, with teacher self-efficacy being a stronger correlate than emotion regulation. The findings offer significant implications for English as a Foreign Language (EFL) teachers.

## Introduction

When considering student learning, various factors including teachers’ emotions play a critical role in influencing student performance and achievement (King and Chen, 2019; Fathi et al., 2021). Negative teacher emotions are believed to impede learning whereas positive ones are facilitative (Pekrun, 1992). The literature has widely shown that teacher’s wellbeing can improve deep learning and establish positive emotional and social development among students (Buettner et al., 2016). Teachers’ psychological wellbeing and their social-emotional capacity are reported as key features that foster emotional and social learning activities (e.g., responsiveness, emotional support, and sensitivity) in the class (Buettner et al., 2016). Given that teaching is highly anxiety provoking (Von Münchhausen et al., 2021), teachers are under a lot of work pressure and at risk of poor social-emotional wellbeing. Long-term work stress and anxiety may lead to burnout, which significantly influence teachers’ mental and physical health, and, in turn, impair their students’ mental and physical health (Fathi et al., 2021). Thus, developing social-emotional learning inside the classroom would be difficult if we don’t give full attention to teachers’ own social-emotional wellbeing.

The competence to retain one’s wellbeing and react flexibly to career problems is identified as an important competence for instructors. Previous studies have substantiated this claim, with a growing number of studies in the domains of teacher wellbeing over the last decade (e.g., Collie et al., 2015). Investigations in this line of research have indicated that teachers who tend to be more engaged, persistent, intrinsically motivated, and feel more competent would report a higher level of psychological wellbeing and would exhibit higher quality degree of performance in the classroom (Deci and Ryan, 1985). According to Huppert (2009), the concept of psychological wellbeing pertains to individuals evaluation of their sustained happiness, mental health, and pleasure and it refers to a number of psychosocial factors such as job satisfaction. Sisask et al. (2014) defined teacher psychological wellbeing as teachers’ satisfaction with their workplace, which can influence their professional performance. Teacher psychological wellbeing is deemed to have a close relationship with stress and job satisfaction (Capone and Petrillo, 2020; Fathi et al., 2021).

According to Burić and Moè (2020), teachers’ emotions affect both teachers’ and students’ motivation, cognition, and behaviors. Educational setting is replete with a pool of different emotional demands asking for those teachers who have the competency and capability to manipulate and manage both negative and positive emotions related to teaching which are likely to affect their instruction and wellbeing (Heydarnejad et al., 2021). Generally, emotion regulation refers to how a person tries to influence the emotions he/she has, when to have them, and how to express these emotions (Gross, 1998). According to Gross and Thompson (2007), emotion regulatory acts may be observed as having their primary effect at various points in the emotion generative process. The language teacher emotion regulation refers to a set of strategies that language teachers employ to regulate their emotions (Heydarnejad et al., 2021). In order to have effective teaching, teachers often need to regulate their emotions (Taxer and Gross, 2018). Investigations in this area have reported that teachers use various emotion regulation strategies in order to regulate their classroom emotions (Taxer and Gross, 2018). As Gross (2002) asserted, teachers determine the emotion regulation strategies to apply inside the classroom depending on their emotions. Negative emotions, for instance, could affect teachers’ instructional behaviors (Frenzel et al., 2009b), or job motivation and their wellbeing (Burić and Frenzel, 2019). Nonetheless, the negative effect of these emotions can be alleviated via employing successful emotion regulation techniques. It is widely agreed that decreasing negative emotions and enhancing positive emotions play a major role in effective teaching (e.g., Frenzel et al., 2009a). Research in educational contexts has indicated that teachers often apply emotion regulation in aid of hedonic goals (psychological health) relating to wellbeing (see Haeussler, 2013). Therefore, emotion regulation strategies could be considered as an important teaching proficiency affecting teachers’ psychological wellbeing and a myriad of classroom outcomes. However, teachers sometimes opt to suppress or neglect their emotions as workplace and power relations in schools may not assign much freedom to instructors, and in turn, affect their articulation of intense emotional suffering (Keller et al., 2014; Taxer and Gross, 2018).

As the third variable under investigation of the present study, self-efficacy refers to ones’ beliefs and perceptions toward their ability in producing particular consequences in specific settings (Bandura, 1997). Bandura (1993) asserted that persons’ perceptions about their competencies influence their anxiety or competence in addressing challenging and demanding conditions, which in turn would affect their psychological wellbeing. Teacher sense of efficacy, more specifically is conceptualized as teachers’ self-appraisal regarding their instructional capability in achieving aspired consequences in the educational contexts (Tschannen-Moran and Hoy, 2001). It can be claimed that one of the most pivotal features that influences both student motivational beliefs and teachers’ instructional quality is teacher self-efficacy. It is well documented that self-efficacious teachers show more willingness to use advanced instructional methods (Czerniak and Lumpe, 1996), and work with struggling learners more persistently (Gibson and Dembo, 1984). Other researchers have shown that instructors with greater self-efficacy are more enthusiastic about teaching (Kunter et al., 2011), experience less work burnout, and experience lower degrees of stress and anxiety than instructors with low self-efficacy (Skaalvik and Skaalvik, 2010).

The influential role that teachers play is more crucial in the context of English language learning where quality communication, good rapport and interaction between students and teachers are crucial because of the nature of the language itself, as both the subject of learning and its means (Pishghadam et al., 2019). English as a Foreign Language (EFL) teachers need to hold constructive and positive perceptions toward themselves, their capabilities in teaching, the instructional processes, and their students in order to play their essential part. This is important because EFL teachers might reduce their degree of effective teaching as well as work engagement unintentionally when they have negative perceptions toward themselves and their job (Greenier et al., 2021). Even though many studies regarding teacher wellbeing factors exist in the literature (e.g., Talbot and Mercer, 2018; Greenier et al., 2021; Li, 2021), the simultaneous interconnections among teacher psychological wellbeing, self-efficacy, and emotion regulation have not been dealt with in the EFL contexts. Therefore, the current study attempted to investigate how teachers’ emotion regulation and self-efficacy would be associated with their psychological wellbeing in the Iranian EFL context. It is worth noting that English in Iran is taught in both public sector (i.e., schools and universities) and private sector (e.g., private language institutions). Private sector teachers are assigned relatively further freedom in terms of using textbooks created by the natives, number of hours dedicated to English teaching, and having less crowded classrooms. These teachers are usually required to use English as the medium of their instruction in the classroom. In contrast, public sector teachers have to follow the policies of the government, teach pre-determined localized textbooks, and have more crowded classrooms, but they can use the first language in their teaching. Overall, teachers of both sectors should generally stick to Islamic perspectives and governmental policies which are reflected in the syllabi, methodology, and cultural norms, leading to less freedom of English teachers in Iran (Mirhosseini and Khodakarami, 2015).

## Literature Review

Teaching is usually seen as a caring occupation requiring a powerful sense of morality, devotion, as well as responsibility for pre-service teachers. This social requirement tends to pose great impact on instructors’ emotion regulation (Pekrun, 1992). Teachers continually find themselves in some contexts in which they should provide optimal answers to educational environment demands and of the students (Manasia et al., 2020). Therefore, emotions might be considered more intense in teaching than other occupations. Research has substantiated that teachers have influential roles in children’s scholastic lives, and teachers wellbeing is inextricably associated with children’s academic performance and socioemotional adjustment (Roth et al., 2007). More specifically, teacher wellbeing has significant impacts on the level of students’ outcomes, achievement, engagement, and motivation (Mercer and Gregersen, 2020). Accordingly, it is immensely important to take into consideration teachers’ wellbeing. Given that language teaching is among the occupations with the highest degrees of stress, teachers bear many negative emotions and stress in their work environment (Mercer and Gregersen, 2020). As such, the investigation of EFL teachers’ psychological variables has been the subject of significant number of studies recently (e.g., Talbot and Mercer, 2018; Kong, 2021; Liu et al., 2021; Chu and Liu, 2022; Liu and Chu, 2022). For example, Liu and Chu (2022) carried out a quantitative study to examine the construct of resilience among Chinese EFL teachers. Recruiting 658 Chinese senior high school EFL instructors and using facto analyses, the researchers verified a three-factor structure of teacher resilience including tenacity, optimism, and coping style. Furthermore, the amount resilience among EFL teachers was reported to be moderate to high.

The findings from Yin et al. (2018) study revealed that school-level emotional work requirements of teaching were positively linked to teachers’ depression and anxiety. It is reported that long-term stress and anxiety inevitably affect teachers’ mental health, decrease their physical preparedness, deplete their’ enthusiasm, and lead to job burnout (Schaufeli et al., 1993). Thus, it seems warranted to postulate that the performance of language teachers in their teaching activities is affected by some psychological traits, including psychological wellbeing and emotion regulation. Research in wellbeing has changed steadily in recent years (Capone and Petrillo, 2020). There has been a shift from the distress symptoms research to the exploration of resources, and personal strengths from a positive psychology perspective which focuses on ways of building and monitoring (Keyes, 2007; Capone and Petrillo, 2020). Positive psychology aims to elevate the development and capacity of one by focusing on the individual’s strength (Kern et al., 2016). Rooted in positive psychology, teacher wellbeing has recently gained huge momentum in teacher education (Aulén et al., 2021). Wellbeing constitutes a concept which is widely used in its diverse interpretations and forms. They include an amalgamation of positive affect, low levels of negative affect, and life satisfaction (Beiser, 1974), or personal growth and mastery experiences (Tay and Diener, 2011). The need to relate both functioning well and feeling good in theoretical approaches of wellbeing has been emphasized (Kern et al., 2016). The concept of wellbeing has been studied in two forms: psychological wellbeing and subjective wellbeing (Kállay and Rus, 2014). Subjective wellbeing is defined as the absence of negative affect, the presence of positive affect, and life satisfaction (Myers and Diener, 1995). Emotional and cognitive types are two general components of subjective wellbeing; whereas the emotional element is concerned with negative and positive affect, the cognitive element is linked to the life satisfaction of individuals (Schimmack et al., 2002). On the other hand, psychological wellbeing maintains a balance between the perception of negative and positive emotions (Ryff, 1989). It also reveals individual’s potential and their tendency to live life to the full (see Forgeard et al., 2011).

It is postulated that the role of psychological wellbeing has a more prominent role in causing than predicting variance in performance. In other words, individuals with higher levels of wellbeing exhibit more resilience, better psychological resources, and are more capable of coping with issues (Obrenovic et al., 2020). Similarly, Lyubomorsky et al. (2005) stated that higher psychological wellbeing is strongly related to many positive dimensions in terms of professional career and personal life. With regard to teachers, their psychological wellbeing has a salient role in influencing their mental health and performance (Roffey, 2012). This is in line with Greenier et al.’s (2021) study which indicated that teachers’ psychological wellbeing is a strong predictor of work engagement among British and Iranian English teachers. This outcome can be interpreted in light of the importance of teacher emotions that lie at the core of teaching (see Lee et al., 2016). Emotions are considered multidimensional phenomena which comprise cognitive, expressive, affective, psychological, and motivational components (see Frenzel and Stephens, 2013). In the domain of teacher emotion, the concentration is mostly on the affective components of emotions, including feelings of nervousness and unease in anxiety (e.g., Keller et al., 2014) or on caring and love (Sutton and Wheatley, 2003). Teachers’ emotions are considered in the current investigation since teaching is not only linked to cognitive experiences but it also involves emotional practices and teachers should regulate their emotions to have effective teaching. As Frenzel et al. (2009a) argued, emotions are integral part of individuals’ teaching practice and should not be neglected.

Teaching is regarded as a rewarding but stressful and emotionally charged job because of difficulties in working with diverse learners and a lack of social support (Richards, 2020). It is reported that one’s stress in the workplace can interfere with their motivation and abilities to perform well in their profession (Sandilos et al., 2015). Moreover, chronic exposures to stressors could culminate in social, physical, and psychological challenges, which then lead to harsh situations like burnout (see Friedman-Krauss et al., 2014). Because of the emotional and sensitive nature of their occupation, instructors are at highly heightened risk of emotional exhaustion which is perceived as a main aspect of burnout (Chang, 2013).

According to Gross (1998), one group of coping strategies which aid people in managing stressors through alleviating the unpleasant emotions is known as emotion regulation. Thompson (1994) highlighted that emotion regulation pertains to intrinsic or extrinsic processes that are responsible for altering, assessing, or controlling emotional reactions, more particularly their temporal and intensive feature in order to accomplish one’s objectives. Generally, emotion regulation encompasses two different strategies, namely emotional suppression and cognitive reappraisal (Gross and John, 2003). In case an anxiety-provoking condition occurs, those who utilize cognitive reappraisal strategies tend to reassess the stressor’s meaning and try to alter their attitude (Gross and John, 2003). Conversely, those who use suppression strategies hinder their emotional expression while encountering emotional agony or discomfort (see Gross and John, 2003). Researchers found that teachers often reduce their aversive, unpleasant emotions or increase their positive emotions by utilizing emotion regulation strategies (Chang, 2009). Teachers’ emotion regulation can protect teachers against burnout, elevate the quality of teaching, and result in increasing the learning quality of learners (Chang, 2009). EFL teaching has its own specific set of distress and disappointment which can result in teachers’ stress and burnout. One strategy for mitigating such frustrations is emotion regulation (Fathi et al., 2021). By using emotion regulation strategies, language teachers can modulate responses that are triggered by emotional demands (Heydarnejad et al., 2021).

Although the literature in language education has witnessed a growing interest in the research of learners’ emotion regulation and their emotional experiences in the learning contexts (Zhang et al., 2021), little is known about language teachers’ emotional regulation. Reviewing the existing literature, it is also worth mentioning that despite the surge of investigations in other fields, researchers in the domain of L2 teaching seem to lag behind researchers in the mainstream education in their effort to explore emotional regulation variable as a plausible correlate of teacher psychological wellbeing. Fathi et al. (2021) carried out a study to investigate the mediating role of teacher emotion regulation in affecting the relationship among, teacher self-efficacy, teacher reflection, and burnout among Iranian EFL teachers. Their outcomes demonstrated that emotion regulation and burnout were substantially tied. Moreover, teachers’ self-efficacy was positively predicted emotion regulation. Comparing British and Iranian English instructors, Greenier et al. (2021) found that emotion regulation of both groups significantly predicted work engagement, suggesting that language teachers who regulate their feelings tend to become cognitively, psychologically, and emotionally engaged in their instructional practices. As they can successfully use the extrinsic and intrinsic processes to monitor, modify, and evaluate their emotional reactions to accomplish their goals, they make further efforts to enhance the level of their teaching activities and become more energetic in their professional career. In addition to developing and validating the Language Teacher Emotion Regulation self-report instrument, Heydarnejad et al. (2021) compared EFL university and high school teachers use of emotion regulation strategies in their workplace. Findings showed that EFL university teachers were more successful in employing emotion regulation strategies at their workplace than EFL high school teachers. EFL high school teachers mostly attempt to suppress or neglect their experienced emotions and avoid challenging emotional situations. Furthermore, they argued that EFL university teachers who had higher education highly appreciated emotion regulation strategies grounded in self-awareness, reasoning, self-regulation, and thinking skills.

The increasing interest in the construct of self-efficacy can be seen over the last decade (e.g., Fackler et al., 2021; Holzberger and Prestele, 2021). According to social cognitive theory, teacher efficacy may be conceptualized as teachers’ attitudes about their capabilities in making a positive influence on their student learning (Bandura, 1986). Based on this theory, teacher self-efficacy can be defined as teachers’ beliefs about their competence and ability in planning, organizing, and implementing practices which are essential to achieve particular academic objectives (Skaalvik and Skaalvik, 2010). In the past two decades, research has demonstrated that teacher efficacy is closely linked to indicators of teachers wellbeing including emotional exhaustion, job satisfaction, and work engagement (Burić and Kim, 2021). For instance, Skaalvik and Skaalvik (2010) demonstrated that self-efficacy affects job satisfaction. Moreover, the findings from Greenier et al.’s (2021) study evinced that a negative correlation existed between teacher self-efficacy and burnout. Meanwhile, teacher efficacy is significantly affected by teachers’ attitudes toward their specific teaching context, demands of their teaching tasks, and assessments of the support and resources available to them (e.g., Bandura, 1997; Tschannen-Moran and Hoy, 2001).

As far as teacher self-efficacy is concerned, it is believed that efficacious instructors tend to use more positive criticism with those students who constantly make mistakes (Tschannen-Moran and Hoy, 2001), as well as inspire more success and motivation in their students than less self-efficacious instructors (e.g., Caprara et al., 2006). Likewise, Hajovsky et al. (2020) reported that teachers with higher self-efficacy beliefs tended to have higher degrees of closeness and lower level of conflict with their students across different grades. They believed teachers who have positive belief about their competence and ability in teaching and managing classroom behavior are likely to carry out activities that lead them to establish supportive and secure relationships with their students. Tschannen-Moran and Hoy (2001) highlighted that teacher self-efficacy is an influential variable that can determine success or failure in different aspects of education. Moreover, teachers with high self-efficacy might make effort and display better organizing and planning skills (Pajares, 1992). Teacher self-efficacy has also drawn attention of some researchers in EFL context in recent years (Fathi et al., 2021).

In another study, Safari et al. (2020) indicated EFL teacher self-efficacy is a positive predictor of professional development. It is worth mentioning that self-efficacy predicted professional development more strongly than reflective thinking and job satisfaction. In accordance with the association between instructor psychological wellbeing and self-efficacy, Cansoy et al. (2020) found that teacher psychological wellbeing and teacher self-efficacy were significantly and positively tied. Moreover, self-efficacy was a positive predictor of teacher psychological wellbeing. Using a criteria-based review approach, Zee and Koomen (2016) investigated the role of teacher self-efficacy in teachers’ psychological wellbeing and the students’ academic adjustment. Findings showed that teacher self-efficacy is positively correlated with students’ academic adjustment, and variables underlying teachers’ psychological wellbeing such as job satisfaction, commitment, and personal accomplishment. Additionally, teacher self-efficacy was negatively linked to burnout. Examining the association between mental health and teacher self-efficacy among 742 teachers, Von Münchhausen et al. (2021) found that teacher self-efficacy and mental health were significantly and moderately correlated. Furthermore, teacher self-efficacy was liked to positive emotions and work-related psychological resistance. Also, the higher teacher self-efficacy, the more improvement in life satisfaction as well as distancing ability was reported. Less teacher self-efficacy was accompanied by reduced social support experience.

Although researchers in different contexts have reported findings regarding the relationship between teacher self-efficacy and teacher psychological wellbeing (e.g., Cansoy et al., 2020), there is still a dearth of investigation among EFL teachers. The only study that has touched this area is Fathi et al. (2020) study, which tried to uncover the role of teacher self-efficacy and psychological wellbeing of Iranian EFL teachers. The results revealed that both variables predicted psychological wellbeing. However, teacher self-efficacy was a more powerful predictor of psychological wellbeing. While previous studies have been valuable in revealing the role of teachers’ self-efficacy and emotion regulation in teaching EFL, no study, to the best of our knowledge, has ever considered these two variables simultaneously in relation to teacher psychological wellbeing, Furthermore, teacher emotion regulation has been dealt with rarely in the EFL context (e.g., Fathi et al., 2021). Moreover, EFL teachers’ psychological wellbeing has not received much attention. Therefore, a significant gap exists in the literature with respect to understanding how teachers’ emotion regulation strategies and their efficacy can be related to their psychological wellbeing. To fill these research gaps with the purpose of encouraging and supporting more comprehensive explorations of teacher health and wellbeing, we aimed to examine the role of teachers’ self-efficacy and emotion regulation in predicting EFL teachers’ psychological wellbeing.

## Materials and Methods

### Participants

Concerning the aim of this study, 276 Iranian English teachers participated in this survey. The participants were practicing EFL teachers who were selected from various cities, provinces, and areas in Iran through convenience sampling. The teachers were both male (*N* = 113) and female (*N* = 163) in-service English instructors who volunteered to complete the battery of electronic questionnaires. Their age varied from 20 to 51 (*M* = 25.35, *SD* = 7.83) and their teaching experience ranged from 1 to 28 years (*M* = 5.74, *SD* = 2.01). The vast majority of the participants had studied English majors in colleges or universities. All of the teachers had experienced teaching EFL in schools or universities.

### Instruments

#### Teacher Self-Efficacy Scale

EFL teachers’ level of self-efficacy was measured by the Teachers’ Sense of Efficacy Scale (TSES) validated by Tschannen-Moran and Hoy (2001). TSES consists of 24 items which measure teachers’ perceptions of their competence in using appropriate strategies, engaging their students, and classroom management. Each item is measured on a 5-point Likert scale ranging from 1, “nothing,” to 5, “a great deal.”

#### Emotion Regulation Scale

EFL teachers’ emotion regulation was measured using the 10-item self-report scale developed by Gross and John (2003). This questionnaire, which measures participants’ tendency to regulate their emotions, includes two components: (a) Cognitive Reappraisal and (b) Expressive Suppression. Each response is evaluated on a 7-point Likert scale, varying from 1 (strongly disagree) to 7 (strongly agree).

#### Psychological Wellbeing Scale

To measure teachers’ degree of psychological wellbeing, the Index of Psychological Wellbeing at Work designed by Dagenais-Desmarais and Savoie (2012) was employed in this research. This 25-item scale includes five sub-scales: Desire for Involvement at Work, Interpersonal Fit at Work, Feeling of Competency at Work, Thriving at Work, and Perceived Recognition at Work. Every item is measured on a 6-point Likert scale (from 0 = Disagree to 5 = Strongly Agree).

### Procedure

To achieve the objectives of this research, the sample of participants were requested to fill out the online survey constituting a battery of valid measures for each of the three constructs. The online survey was constructed by inserting the items of the questionnaires using the Google Docs application. Then the link of the online survey was shared with Iranian English teachers from various parts of the country. Before responding the items of the questionnaires, the teachers were provided with explanations on how to fill out the Google Docs forms. Then they were notified that their filled questionnaires would be used for research purposes and the confidentiality of gathered information would be assured.

## Data Analysis and Results

The data were analyzed using Statistical Package for Social Sciences (SPSS 22) and Analysis of Moment Structures (AMOS 21) software. Apart from descriptive statistics calculations, confirmatory factor analyses (CFAs) and Structural Equation Modeling (SEM) were performed. A robust multivariate procedure, SEM is employed to verify hypothesized structural theories. CFA was used to check the validity of the latent constructs prior to examining the structural model (Hair et al., 1998).

As the first step of data analyses, missing values, outliers, and normality of the data were investigated via data screening. Missing data were addressed employing an expectation– maximization algorithm where missing data are substituted by random values (Kline, 2011). Univariate and multivariate outliers were checked through standard scores and Mahalanobis D^2^, respectively. Also, the kurtosis and skewness values falling outside the range of –1 to + 1 were considered non-normal. Given these initial screening, all the outliers and non-normal values were determined and discarded, resulting in 269 valid cases for further analyses.

The measurement models for the three latent constructs were examined through running CFAs and fit indices were considered to verify their validity (Kline, 2011). More precisely, Chi-square divided by degree of freedom (χ^2^/df), Comparative Fit Index (CFI), Tucker–Lewis Index (TLI), and Root Mean Square Error of Approximation (RMSEA). Following Tseng and Schmitt (2008), we regarded a model to be fit in case χ^2^/df < 3, CFI and TLI > 0.90, and RMSEA < 0.08.

As measurement models failed to demonstrate adequacy to the data, some changes were made on the models. These changes involved the omission of items with low loadings from the questionnaires including: two items from teacher self-efficacy measure, one item from emotion regulation measure, and two items from psychological wellbeing measure. The modified models indicated good fit (see Table 1). Concerning the reliability, all the obtained coefficient alphas for the measures exceeded 0.70, showing the appropriateness of the internal consistencies (see Table 1). Following that, descriptive statistics and correlation coefficients of the constructs were calculated (see Table 2).

**TABLE 1:** Measurement model of the latent constructs.

	χ^2^	Df	χ^2^/df	CFI	TLI	RMSEA	α
Self-efficacy	35.12	18	1.95	0.96	0.95	0.05	0.81
Emotion regulation	8.24	4	2.06	0.94	0.93	0.07	0.73
Wellbeing	13.85	8	1.73	0.98	0.98	0.03	0.89

**TABLE 2:** Descriptive statistics and correlations.

	*M* (SD)	1	2	3
(1) Self-efficacy	4.12 (1.21)	1.00		
(2) Emotion regulation	3.42 (0.91)	0.22*	1.00	
(3) Wellbeing	3.78 (1.13)	0.56**	0.34**	1.00

*p < 0.05; **p < 0.01.

### Model Testing

**FIGURE 1:**
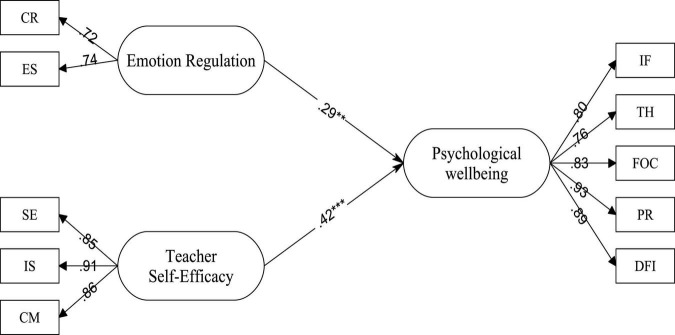
The final model of teacher self-efficacy, emotion regulation, and teacher psychological wellbeing. *p < 0.05; **p < 0.01; ***p < 0.001.

The hypothesized, structural model was tested using AMOS 21 with the maximum likelihood procedure and variance-covariance matrices as the input. The results indicated the significance of path coefficients (*p* < 0.05) as well as the adequacy of the fit indices. SEM results confirmed all the hypotheses in the proposed model (see Figure 1). For the meaningfulness of data interpretations, effect size (ES) (Cohen’s *f*^2^) was computed for the latent constructs (Table 3).

**TABLE 3:** Standardized parameter estimates for the structural model.

	*R* ^2^	*f* ^2^
(1) Self-efficacy	0.17	0.20
(2) Emotion regulation	0.08	0.08

As illustrated in Figure 1, emotion regulation had a small effect on teachers’ psychological well-being (β = 0.290.29, *R*^2^ = 0.080.08, *f*^2^ = 0.080.08, small effect size). Additionally, it was found that teacher self-efficacy was a stronger predictor of psychological well-being (β = 0.420.42, *R*^2^ = 0.170.17, *f*^2^ = 0.200.20, medium effect size).

## Discussion

In order to enlighten teachers’ psychological wellbeing and its antecedents in EFL contexts, the current study was set to explore the predictive role of self-efficacy and emotion regulation in affecting teacher psychological wellbeing among Iranian EFL teachers. Given that how teachers regulate their negative and positive emotions is regarded a key variable influencing wellbeing of both teachers and their learners (see Chang, 2009), we investigated how teacher emotion regulation contributed to influencing their psychological wellbeing. Also, the effect of teacher self-efficacy on psychological wellbeing was investigated in the hypothesized model. The findings yielded three intriguing results.

First, it was demonstrated that teacher sense of efficacy could substantially predict psychological wellbeing of EFL teachers. This outcome resonates with the findings of a notable body of research highlighting that high degrees of teachers’ self-efficacy are associated with high levels of psychological wellbeing (Zee and Koomen, 2016; Bentea, 2017; Fathi et al., 2020; Lipiñska-Grobelny and Narska, 2021). For instance, Lipiñska-Grobelny and Narska (2021) reported that teachers’ self-efficacy was linked to their psychological wellbeing. In other words, teachers with high self-efficacy had higher degrees of positive emotions and satisfaction and felt lower degrees of negative emotions.

One possible explanation might be that teachers with greater degrees of self-efficacy (e.g., perceptions that they have a substantial effect on students’ development and learning) might be highly inspired and more content with their occupation, which in turn may enhance their psychological wellbeing. This is confirmed by Deci and Ryan (1985) who argued that one’s intrinsic motivation contributes to their psychological wellbeing. Moreover, teachers’ positive attitude toward their teaching might encourage them to foster their classroom management and pedagogic effectiveness via palliating their psychological loads and challenges. It can also be argued that instructors experience a sense of individual achievement and experience less burnout if they are endowed with greater efficacy perceptions and self-assurance in their competencies to teach well and enthusiastically engage their pupils. This is supported by Brouwers and Tomic (2000), who found that teacher self-efficacy has a synchronous positive influence on personal accomplishment and a longitudinal negative influence on depersonalization.

In their longitudinal study, Fernet et al. (2012) also revealed that changes in instructors’ self-efficacy had negative associations with alteration in their degrees of depersonalization and emotional exhaustion and positive relationships with changes in their personal accomplishment. As a result, instructors with high self-efficacy might feel lower degrees of emotional exhaustion and higher levels of commitment and satisfaction, eventually prompting them to eagerly continue their job. Similarly, other researchers have reported that teacher self-efficacy is a key factor influencing teachers’ psychological wellbeing, which enhances the degrees of job satisfaction and teaching commitment and reduces the degrees of teaching stress (e.g., Jepson and Forrest, 2006; Zee and Koomen, 2016: Fathi et al., 2021).

Second, it was found that emotion regulation was a significant predictor of teacher psychological wellbeing. This finding is supported by numerous scholars who have highlighted the importance of teachers’ emotion regulation in classroom contexts and its impacts on teacher wellbeing (Sutton, 2004; Yin, 2016). It is likely to argue that instructors’ coping strategies might reinforce the relationship between emotion regulation and psychological wellbeing. For instance, instructors that employ more appropriate emotion regulation strategies may experience less apprehension from disorganized learning settings and students’ misbehavior since they are capable of handling stressors that occur in the classroom. Therefore, the sense of capability may cause teachers to experience higher level of psychological wellbeing, which helps them to improve their potential satisfaction with their occupation and their classroom practice. On the other hand, when teachers are not capable of regulating their emotions, they cannot manage to deal with the challenges in the classroom; thus, they might perceive their occupation as emotionally draining. Moreover, teachers who are able to self-regulate their emotions can form strategies compatible with their psychological conditions and establish a close and warm relationship with their students. Therefore, teachers are more likely to experience satisfaction and joy with their job and enrich their personal growth as they reach a strong mental health state. This interpretation is consistent with research outcomes of Han and Wang (2021) which showed that instructors’ efficacy perceptions were associated with their emotional and physical engagement in teaching activities.

Lastly, we notably found that although both constructs had a significant impact on psychological wellbeing of EFL teachers, self-efficacy surpassed emotion regulation in affecting teachers’ psychological wellbeing. It might be posited that teachers’ high level of competence and efficacy regarding their teaching capability might decrease their degrees of apprehension, frustration, and depression. This interpretation aligns with the results of some prior studies that recognized teacher self-efficacy as a negative correlate of their disengagement and burnout (Fathi et al., 2021). From this perspective, higher levels of self-efficacy in teaching might be linked to greater job satisfaction and positive job desires. That is, instructors with greater levels of teaching efficacy might become more self-assured in employing emotional regulation techniques if they encounter difficult and challenging contexts; consequently, they experience less anxiety in their occupation than teachers who have lower levels of self-efficacy. As a result, teachers’ positive emotions (i.e., less anxiety and further job satisfaction) can enhance their psychological wellbeing as well as optimal functioning, thereby leading them to further dedication to teaching activities and work engagement. This is verified by Aiello and Tesi (2017) who portrayed that instructors’ wellbeing in classroom contexts is significantly correlated with their engagement levels. Greenier et al. (2021) also maintained that work engagement in learning context could be remarkably enhanced by their psychological wellbeing.

## Conclusion

Taken together, the present study identified two potential predictors of EFL teachers’ psychological wellbeing namely self-efficacy and emotion regulation. It was revealed that both variables predicated teachers’ psychological wellbeing significantly, with teacher self-efficacy having a higher share of variance. This study adds valuable information to the literature by revealing the importance of teacher variables in predicting their psychological wellbeing in their workplace. Lastly, this study furnished some indications that EFL teachers who tend to be less efficacious and unfamiliar with emotional regulation strategies are at risk of job exhaustion. Altogether, the present study opens the path to further research, which could focus more deeply on the specific impacts of various emotion regulation strategies and different types of self-efficacy when considering their increase in teacher wellbeing as the most significant target to improve.

Also, the results from this research might have several practical implications for EFL teacher educators and researchers. To promote teacher wellbeing, EFL teacher educators should provide training or mentoring via professional development programs on how to determine different stressors and aid instructors in recognizing how they influence teachers’ emotions and emotion regulation in the classroom. The administrators might also foster a positive and open learning context to prompt EFL instructors to verbalize real emotions as well as learning to effectively re-assess diverse classroom contexts. If practitioners view their job situations and environments as more pleasant, they may eagerly find techniques to overcome their emotional challenges while teaching. Based on the outcomes of this research, it is crucial to aid EFL practitioners in forming self-efficacy or competence, which was identified as a powerful antecedent of their psychological wellbeing in the EFL context. This might be approved by offering teachers learning opportunities to bridge the gaps regarding their instructional body of knowledge. Moreover, administrators or principals could assign further power to EFL instructors to build more favorable perception toward their teaching competencies.

Besides enhancing teaching abilities, treatment activities are required to deal with the possible stressors that instructors experience in their work contexts and techniques for regulating their emotions while facing challenging situations. They should also get ready to deal with different stressors within their workplace, including interactions with their students, colleagues, administrators, and fatigue or tension from large and chaotic classes. EFL teacher educators should take the initiatives to design training and professional development programs to raise awareness of the negative emotions and stressor which may occur in the classroom, and how teachers can handle them. By participating in these programs, teachers could benefit from reflecting on the various ways in which they manage and direct their emotions so as to accomplish classroom aims and their efficacy within context. Investigating practical ways that help EFL teachers to detect and reframe their perceptions about teaching and emotion regulation could be a worthy direction for further research and implications.

## Limitations

The results obtained from the present research have several limitations. Even though this study has some notable impacts on our understanding and knowledge of how teacher emotion regulation and their beliefs are related to their psychological wellbeing, and how they affect their teaching quality, it has still some limitations in its sample size and design. First, even though we recruited a relatively big number of EFL instructors in Iran, still bigger data is needed to increase the generalizability of the study. It might be argued that those instructors who were struggling with psychological problems might have decided not to partake in the research because either they did not wish to reveal their psychological challenges or they had low energy to fill out and return the survey. Second, because of the nature of survey research, it seems difficult to find what emotion regulation strategies instructors might employ to manage their emotions in various occasions. Although instructors are aware of their own emotions and beliefs, it is recommended that further research employ several elicitation techniques (e.g., reflective journals and interviews) to triangulate the findings. Moreover, future research is needed to understand and better explain causal relationships between teacher self-efficacy and emotion regulation. Future longitudinal or intervention studies may shed more light on the causality inferences among the constructs. Therefore, longitudinal studies are needed to make any causal claims in conjunction with the associations among the variables. Also, further research is required to identify whether these factors affect more enduring outcomes.

It would be interesting if future studies could investigate whether the correlations between (a) teacher efficacy and their psychological wellbeing, (b) emotion regulation and teachers’ psychological wellbeing could extend to student outcomes and classroom practices. Future studies are also recommended to explore teachers’ beliefs (teaching efficacy and competence) and perceptions toward their work environments, and its relation to teachers’ psychological load including job-related emotional exhaustion, depression, and general perceived stress.

## Data Availability Statement

The data analyzed in this study is subject to the following licenses/restrictions: The raw data supporting the conclusions of this article will be made available by the authors, without undue reservation. Requests to access these datasets should be directed to the corresponding author.

## Ethics Statement

The studies involving human participants were reviewed and approved by the University of Kurdistan. The patients/participants provided their written informed consent to participate in this study.

## Author Contributions

All authors were equally involved in designing the research, topic development, data collection, data analysis, writing drafts, and final editing.

## Conflict of Interest

The authors declare that the research was conducted in the absence of any commercial or financial relationships that could be construed as a potential conflict of interest.

## Publisher’s Note

All claims expressed in this article are solely those of the authors and do not necessarily represent those of their affiliated organizations, or those of the publisher, the editors and the reviewers. Any product that may be evaluated in this article, or claim that may be made by its manufacturer, is not guaranteed or endorsed by the publisher.
